# Endolysin EN572-5 as an alternative to treat urinary tract infection caused by *Streptococcus agalactiae*

**DOI:** 10.1007/s00253-023-12949-8

**Published:** 2024-01-08

**Authors:** Maria Kajsikova, Michal Kajsik, Lucia Bocanova, Kristina Papayova, Hana Drahovska, Gabriela Bukovska

**Affiliations:** 1 Department of Genomics and Biotechnology, Institute of Molecular Biology SAS, Dubravska cesta 21, 845 51 Bratislava, Slovakia; 2 Comenius University Science Park, Ilkovicova 8, 841 04 Bratislava, Slovakia; 3 Department of Molecular Biology, Faculty of Natural Sciences, Comenius University in Bratislava, Ilkovicova 6, 841 15 Bratislava, Slovakia

**Keywords:** *Streptococcus agalactiae*, UTI GBS, Endolysin, Bacteriolytic activity, EN572-5

## Abstract

**Abstract:**

*Streptococcus agalactiae* (Group B Streptococcus, GBS) is an opportunistic pathogen causing urinary tract infection (UTI). Endolysin EN572-5 was identified in prophage KMB-572-E of the human isolate *Streptococcus agalactiae* KMB-572. The entire *EN572-5* gene was cloned into an expression vector and the corresponding recombinant protein EN572-5 was expressed in *Escherichia coli* in a soluble form, isolated by affinity chromatography, and characterized. The isolated protein was highly active after 30 min incubation in a temperature range of − 20 °C to 37 °C and in a pH range of 5.5–8.0. The endolysin EN572-5 lytic activity was tested on different *Streptococcus* spp. and *Lactobacillus* spp. The enzyme lysed clinical GBS (*n* = 31/31) and different streptococci (*n* = 6/8), and also exhibited moderate lytic activity against UPEC (*n* = 4/4), but no lysis of beneficial vaginal lactobacilli (*n* = 4) was observed. The ability of EN572-5 to eliminate GBS during UTI was investigated using an in vitro model of UPSA. After the administration of 3 μM EN572-5, a nearly 3-log decrease of urine bacterial burden was detected within 3 h. To date, no studies have been published on the use of endolysins against *S. agalactiae* during UTI.

**Key points:**

*• A lytic protein, EN572-5, from a prophage of a human GBS isolate has been identified.*

*• This protein is easily produced, simple to prepare, and stable after lyophilization.*

*• The bacteriolytic activity of EN572-5 was demonstrated for the first time in human urine.*

**Graphical abstract:**

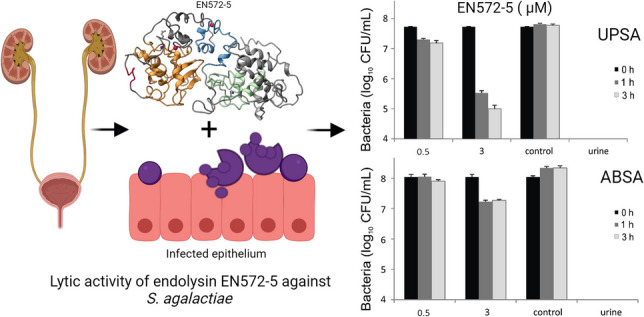

**Supplementary Information:**

The online version contains supplementary material available at 10.1007/s00253-023-12949-8.

## Introduction

Urinary tract infections (UTIs) are one of the most common bacterial infections, affecting 150 million people worldwide each year (Ala-Jaakkola et al. 2022; Flores-Mireles et al. 2015). The most frequent causative agents of UTIs are the uropathogenic *Escherichia coli* (UPEC), *Klebsiella* species, *Enterococcus faecalis*, and *Proteus mirabilis* (Mohanty et al. 2003; Magliano et al. 2012; Medina and Castillo-Pino 2019). Although it is a causative pathogen, *Streptococcus agalactiae* is responsible for approximately 2–3% of all UTIs (Tan et al. 2012; Foxman 2010).

*S. agalactiae*, also known as Group B *Streptococcus* (GBS), is a commensal inhabitant of the human gastrointestinal and genitourinary tracts that can cause various forms of UTI, including asymptomatic bacteriuria (ABU), cystitis, and pyelonephritis (Tan et al. 2012; Ulett et al. 2009). Although both men and women may become infected, UTIs are usually a disease of women. In pregnancy, the detection of GBS in urine (at any count) is considered a risk factor for the vertical transmission of this pathogen to the neonate. Untreated GBS infection in pregnancy is reported to be associated with preterm rupture of the membranes, premature delivery, and early onset neonatal sepsis (Schnarr and Smaill 2008; Lumbiganon et al. 2010; Verani et al. 2010).

ABU-causing *S. agalactiae* (ABSA) strains are able to grow in urine, while some other uropathogenic *S. agalactiae* (UPSA) cannot (Ipe et al. 2016). The predominant GBS serotype in UTIs is serotype III (Sullivan et al. 2017) while the second and third most common serotypes are V and Ia (Ulett et al. 2009; Gherardi et al. 2007; Dobrut et al. 2022).

GBS infections are routinely treated with antibiotics, mainly beta-lactams. However, there have been reports of reduced susceptibility of GBS to beta-lactams, including penicillin (Hiroaki et al. 2019). Resistance of GBS strains to second-line antibiotics, such as erythromycin and clindamycin, is also high, and increasing rates of resistance have been observed in several countries (France, Spain, Italy, China, South Korea) in recent years (Hayes et al. 2020). Vancomycin, the antibiotic of last resort, is employed in cases when patients are allergic to penicillin and second-line antibiotics are ineffective and still remain largely effective (Hayes et al. 2020). An additional consideration is that antibiotic-induced changes in the microbial composition of the intestine or vagina can have a negative impact on human health, including reduced microbial diversity and changes in the functional attributes of the microbiota (Patangia et al. 2022).

Given the increasing prevalence of multidrug-resistant bacteria over the last 20 years, it has become necessary to develop new antimicrobial agents to treat infections caused by resistant bacteria. One approach, first tried nearly a century ago and recently revived, is the use of bacteriophages to treat bacterial infections (Miguel et al. 2020). Treatment using phage and phage lytic enzymes (PLEs) has a great potential for eliminating pathogenic GBS (Wong et al. 2022). Therapeutic bacteriophages must be strictly lytic and exhibit high host specificity. However, no lytic phage infecting GBS has been isolated to date, and only temperate ones have been characterized (Bai et al. 2013; Domelier et al. 2009; Furfaro et al. 2020).

Newer, but related treatment strategies make use of phage-derived therapeutics such as peptides and enzymes. Particular attention is given to lysins and endolysins, which degrade the bacterial cell wall. Double-stranded DNA bacteriophages employ a two-component lytic system, including a holin and an endolysin, to release newly assembled phage progeny (Gründling et al. 2001). Endolysin degrades peptidoglycan (PG), a main structural component of the cell wall, at the end of the bacteriophage replication cycle, resulting in bacterial cell lysis and the release of progeny virions from the cell. Many recombinant endolysins have already been expressed, identified, and purified that display strong bacteriolytic activity when applied exogenously (Grabowski et al. 2021). This feature makes them important potential alternatives to antibiotics (Liu et al. 2023a).

The genes encoding endolysins are present in the genomes of lytic and temperate bacteriophages. Endolysins from phages infecting Gram-positive bacteria mostly have a two-domain modular structure, usually composed of an N-terminal enzymatically active domain (EAD) and a C-terminal cell wall–binding domain (CBD), which are linked by a short flexible linker. There can be variations on this arrangement or even multiple EADs (Schmelcher et al. 2012). Endolysins are classified into five different groups depending on the cleavage site of the peptidoglycan cell wall by the EADs: acetylmuramidases, lytic transglycosylases, glucosaminidases, amidases, and endopeptidases (Broendum et al. 2018).

Endolysins targeting *Streptococcus* species are unique due to the distinct types, numbers, and organization of their EADs and CBDs. Only five endolysins originating from *S. agalactiae* prophages have been characterized to date: B30, PlyGBS, λSa1 and λSa2, and EN534-C. The PlyGBS and B30 endolysins have similar domain organizations: CHAP domains at the N-terminus of the protein and a GH25 glycosidase domain in the middle. The C-terminal CBD of PlyGBS is unknown, while B30 has an SH3b CBD (Cheng et al. 2005; Donovan et al. 2006). Prophage endolysins λSa1 and λSa2, which both originate from *S. agalactiae* 263 V/R, contain a similar endopeptidase domain at the N-terminus, but λSa2 has an additional amidase domain at its C-terminus. The CBDs of these enzymes are also different: λSa1 contains a SH3b domain at the C-terminus while λSa2 possesses two copies of a Cpl-7 CBD in the middle (Pritchard et al. 2007). EN534-C endolysin has two terminal catalytic domains, amidase_3 and CHAP, and one central binding domain, LysM (Bocanova et al. 2022). These differences largely determine the strength and spectrum of the enzyme (Wong et al. 2022). The host ranges of these endolysins were also different. PlyGBS can kill multiple strains of GBS, whereas B30 has wider lytic activity, including activity against *Streptococcus* groups A, B, C, E, and G (Pritchard et al. 2004; Cheng et al. 2005). The λSa1 and λSa2 endolysins showed similar lytic activity on *S. agalactiae*, *S. pneumoniae*, and *S. aureus*.

In this study, we characterized a new endolysin, EN572-5, derived from the prophage region of the human clinical isolate *S. agalactiae* KMB-572 (Lichvarikova et al. 2020). We report the in silico analysis, expression, purification, and subsequent characterization of the antibacterial activity of recombinant EN572-5 against *Streptococcus* spp., *Lactobacillus* spp. and UPEC. In addition, we examined the antibacterial effect of EN572-5 against *S. agalactiae* strains in urine under in vitro conditions. The antimicrobial potential of bacteriophage endolysins to eliminate the growth of *S. agalactiae* in human urine has not yet been investigated.

## Materials and methods

### Bacterial strains and growth condition

The bacterial strains used in this study are listed in Table S1a. *S. agalactiae* CCM 6187 (Lehmann and Neumann 1896^AL^, CCM, Brno, Czech Republic) was used as a control strain. *S. agalactiae* clinical strains were isolated from clinical samples of patients at University Hospital (Bratislava, Slovakia) during 2016–2018 and from women during pregnancy screening between the 33rd and 37th week of pregnancy by the Laboratory of Clinical Microbiology, Medirex (Nitra, Slovakia) during 2018 (Lichvarikova et al. 2020). Other streptococci species were obtained from the Collection of Microorganisms of Comenius University (Bratislava, Slovakia) and Comenius University Science Park (Bratislava, Slovakia). All streptococcal strains were cultivated aerobically overnight in Todd Hewitt broth (THB) and on Todd Hewitt agar (THA) for 24 h at 37 °C. *Lactobacillus* strains were obtained from Faculty of Pharmacy CU (Bratislava, Slovakia) and were grown statically in MRS broth at 35 °C. Uropathogenic *E. coli* (UPEC) strains were obtained from the Collection of Microorganisms of Comenius University (Bratislava, Slovakia). *E. coli* XL1 Blue (Novagen, USA) was used for plasmid DNA cloning and cell stock storage, and *E. coli* BL21(DE3) (Novagen, USA) was used as a host strain for the expression of the recombinant endolysin.

### Bioinformatic analysis of EN572-5

The gene (peg. 604) encoding EN572-5 endolysin was identified on the genome of human isolate *S. agalactiae* KMB-572 (ID 5605; database http://pubmlst.org/sagalactiae) in the prophage KMB-572-E (Lichvarikova et al. 2020). The predicted protein sequence of this endolysin was analyzed using phiBiScan (http://www.phibiotics.org/index.php) (Hojckova et al. 2013). The protein domain composition was predicted using the NCBI Conserved Domain Database (CDD) (https://www.ncbi.nlm.nih.gov/Structure/cdd/cdd.shtml). BLASTP was used to search the non-redundant database using the amino acid sequence of endolysin EN572-5 as a query (Altschul et al. 1997). The online tool Phyre2 web portal for protein modeling, prediction, and analysis (Kelley et al. 2015) was used to predict the EN572-5 protein 3D model structure (http://www.sbg.bio.ic.ac.uk/~phyre2/html/page.cgi?id=index).

### Cloning and protein expression

The putative endolysin gene *EN572-5* was amplified by PCR using the primers: EN572 Ami5Glu F (5´-AGCCATATGGAAATCAACACTGAAACAG-3´) and EN572 Ami5Glu R (5´-CTTGTCGACCTAAACTGGCTTTTTAGTC-3´) with the genomic DNA of the *S. agalactiae* KMB-572 strain used as a template. The resulting PCR product was digested with restriction enzymes *Nde*I and *Sal*I, gel purified, and cloned into the *Nde*I and *Sal*I sites of the pET28a^+^ plasmid (Novagen, USA).

The resulting plasmid pET-EN572-5 was transformed into *E. coli* BL21(DE3) cells to express an N-terminally His-tagged EN572-5 protein. An overnight culture was inoculated (1:100) into fresh LB medium containing 100 µg/mL of kanamycin and the cells were grown at 37 °C to an optical density OD_600_ of 0.7. Protein expression was induced by IPTG at a final concentration of 0.4 mM and the culture was further cultivated for 2 h at 30 °C. The cells were harvested by centrifugation at 20,000 × g for 15 min at 4 °C, washed with physiological saline solution (0.9% (w/v) NaCl), and stored at − 20 °C or immediately used. The protein expression was evaluated by SDS-PAGE.

### Purification of EN572-5

The cell pellet was resuspended using 1/10 volume of lysis buffer (50 mM Tris–HCl pH 7.5, 200 mM NaCl, 5 mM imidazole, 10% (v/v) glycerol) supplemented with Protease Inhibitor Cocktail (Sigma-Aldrich), and cells were lysed by sonication on a Soniprep 150 Plus (MSE). Cell debris was removed by centrifugation at 20,000 × g for 30 min at 4 °C. Recombinant EN572-5 was purified by IMAC on HIS-Select Nickel Affinity Gel (Sigma-Aldrich), previously equilibrated with lysis buffer. Affinity chromatography was performed at room temperature. The column with loaded protein was washed with the same buffer. Protein was eluted from the column with 50 mM Tris–HCl pH 7.5, 200 mM NaCl, 500 mM imidazole, 10% (v/v) glycerol. Fractions containing the purified protein were pooled, concentrated on Amicon® Ultra Centrifugal Filters (cut-off 10 kDa) (Merck), and the buffer was exchanged for a storage buffer containing 50 mM Tris–HCl pH 7.5, 200 mM NaCl. The purified protein was stored at − 20 °C. The isolated protein was quantified using the Bradford protein assay. All protein purification steps, and both the purity and quantity of the isolated EN572-5, were monitored by SDS-PAGE.

### Western blot analysis

Proteins separated by SDS–PAGE (10%) were analyzed by Western blotting. After transfer to a nitrocellulose membrane using a Panther semidry electroblotter (Owl), the His_6_Tag sequence was identified by immunoreaction with a His_6_Tag monoclonal antibody and with a Goat anti-mouse IgG alkaline phosphatase conjugate (both from Novagen) as a secondary antibody. A PageRuler Prestained Protein Ladder (#26616, Thermo Scientific) was used as a molecular weight marker.

### Antibacterial activity

#### Turbidity reduction assay

The lytic effect of recombinant EN572-5 was studied on reference strain *S. agalactiae* CCM 6187 (bovine type) and the human clinical isolate *S. agalactiae* KMB-572 (MLST database ID 5605). Bacterial cultures were grown in THB medium at 37 °C for 18 h; these overnight cultures were subcultured (1:100) in THB medium and grown to mid-exponential phase (OD_600_ ~ 0.5). These grown cell cultures were harvested by centrifugation (2500 × g, 15 min, 4 °C). The pellets were washed with ultrapure water and resuspended in sterile 50 mM Tris–HCl, pH 7.0 (or in another specific medium used in particular reaction assays) to an OD_600_ of 0.7, which is equal to ~ 5 × 10^7^ colony-forming units (CFU)/mL.

These bacterial cultures were used as substrates in reactions with 0.5 μM EN572-5 (final concentration). In the control sample, the storage buffer was used instead of the endolysin. Antibacterial activity was measured in 96-well microtitration plates in total volumes of 200 μL at 37 °C. Assays were performed in triplicate and OD_600_ readings were taken every 5 min with 10 s preshaking up to 60 min in an 800 TS absorbance reader (Biotek).

The lytic activity was calculated using Eq. (1) of Oliveira et al. (2015):

1 $$\mathrm{Lytic\;activity }\left(\mathrm{\%}\right)=\frac{\left({r}_{t0}-{r}_{t60}\right)- \left({c}_{t0}-{c}_{t60}\right)}{{c}_{t0}}\times 100$$

where *r* and *c* are the suspension OD_600_ of the reaction and control samples and *t*0 and *t*60 are the samples at 0 min and 60 min, respectively.

The values measured for the control at each time point were subtracted from the values of the sample measured at the same time point. There was not a sample with an assigned 100% lytic activity with which we would subsequently compare the measured values.

#### Lytic activity of different concentrations of EN572-5

To determine the effect of protein concentration on the lytic activity of EN572-5, bacterial cell cultures were incubated with different concentrations of endolysin: 0.01, 0.1, 0.5, 1, 1.5, and 2 μM (final concentrations). The reactions were performed in triplicate. Lytic activity was determined using a turbidity reduction assay and calculated using Eq. (1) as given above.

#### Effects of different factors on EN572-5 lytic activity

A turbidity reduction assay was used to determine the optimal conditions for the lytic activity of EN572-5. The lytic activity was calculated using Eq. (1) as given above. All experiments were performed in triplicate.

The effect of pH on the lytic activity of EN572-5 was evaluated over the pH range 4.0 to 8.0. Bacterial cells were resuspended in 50 mM CH_3_COONa with pH from 4.0 to 6.0 and in 50 mM Tris–HCl pH 7.0 and pH 8.0. The effect of divalent ions on the lytic activity was determined on bacterial cells resuspended in 50 mM Tris–HCl, pH 7.0 supplemented with increasing concentrations of CaCl_2_ (0–20 mM), MgCl_2_ (0–20 mM), and NaCl (0–200 mM). The effect of temperature on the lytic activity was tested using various samples of EN572-5. The purified enzyme was either frozen at − 20 °C or incubated at 6, 25, 30, 37, 40, and 50 °C for 30 min. The cells used as substrates were resuspended in physiological saline solution.

#### Lyophilization

Purified EN572-5 was lyophilized using a CoolSafe Freeze Dryer 55–9 (ScanVac, Denmark). Briefly, 0.1 mL of EN572-5 (1 mg/mL, 18.1 μM) in solution with 50 mM Tris–HCl pH 7.5, 200 mM NaCl was transferred into a clean 1 mL tube and lyophilized under the following conditions: freezing by liquid nitrogen followed by drying at − 50 °C for 2.5 h at 10 µbar atmospheric pressure. The lyophilized protein was stored at − 20 °C. On the 16th and 120th day after lyophilization, the lyophilized protein powder was re-dissolved in 0.1 mL of ultrapure water and tested for lytic activity.

#### Lytic spectrum

Multiple *Streptococcus* spp. strains and uropathogenic strains of *E. coli* and *Lactobacillus* spp. (Table S1a) were used to test the lytic spectrum of EN572-5. Bacterial cells were grown to mid-exponential phase (OD_600_ ~ 0.5) (*Streptococcus* spp. in THB, UPEC in LB medium, *Lactobacillus* spp. in MRS), washed and resuspended in physiological saline solution, and tested for sensitivity to EN572-5 using a turbidity reduction assay. Gram-negative bacteria were not treated with chloroform or EDTA.

### Antimicrobial activity of EN572-5 in urine

#### Detection of genes from the *mae* gene cluster

The sequences of the *mae* gene cluster of *S. agalactiae* ABSA 1014 and *S. agalactiae* UPSA 807 (accession numbers KU061060 and KU061063) (Ipe et al. 2016) were used as a query to determine the presence of the *mae* gene cluster in the genome of selected *S. agalactiae* clinical strains (Lichvarikova et al. 2020) (Table S1a). Sequences were identified and compared using Geneious version 11.1.5 (Biomatters Ltd., Auckland, New Zealand).

#### Growth of *S. agalactiae* in human urine

A human urine growth assay was performed according to Ipe et al. (2016) with modification. Briefly, we used urine from healthy volunteers: female, male, and child with no recent history of UTI or taking of antibiotics (at least one month before collection). The urine was sterilized by filtration through a 0.22 μm filter, stored at 4 °C, and used within 8 h. Selected clinical isolates of *S. agalactiae* (ABSA 797 and UPSA 564) were grown at 37 °C for 18 h in THB medium, then pelleted at 2500 × g at 4 °C for 15 min, washed, and resuspended in PBS pH 7.4. Approximately 3 × 10^2^ CFU/mL of bacterial cultures were inoculated into 200 μL urine in triplicate and cultivated in 96-well microtiter plates at 37 °C with shaking at 250 rpm. A urine sample without bacteria was used as a negative control. Culture growth was monitored at time intervals of 0, 6, 24, 54, and 72 h by colony counting. For each time point, a 1:10 dilution series in PBS pH 7.4 medium was performed and 10 μL from each dilution was dropped on a plate containing solid THB medium and incubated at 37 °C. Bacterial colonies grown after 24 h were counted. The generation time was calculated using Eq. (2)

2 $$G=\frac{t}{n}=\frac{t}{3.3({\text{log}}{N}_{t}-{\text{log}}{N}_{0})}$$

where *G* is generation time (in min or h), *t* is the exponential growth time (in min or h), *n* is number of generations, *N*_*t*_ is the number of cells at time *t*, and *N*_0_ is the initial number of cells.

#### Lytic activity of EN572-5 in urine

*S. agalactiae* KMB-564 and *S. agalactiae* KMB-797 were grown in THB at 37 °C for 18 h, then subcultured (1:100) and cultivated to mid-exponential phase (OD_600_ ~ 0.5). Bacteria were pelleted at 2500 × g at 4 °C for 15 min and washed in PBS pH 7.4. The cells were diluted in human urine (previously filter sterilized with a 0.22 μm filter) to an initial infectious dose of 5.3 × 10^7^ CFU/mL, and treated with 0.5 and 3 μM EN572-5 (resulting concentration). Samples without endolysin and pure urine were used as negative controls. All reactions were performed in triplicates. Lytic activity was monitored at time intervals of 0, 1, and 3 h using the colony counting method given above.

## Results

### Bioinformatics analysis and expression of endolysin EN572-5

**Fig. 1:**
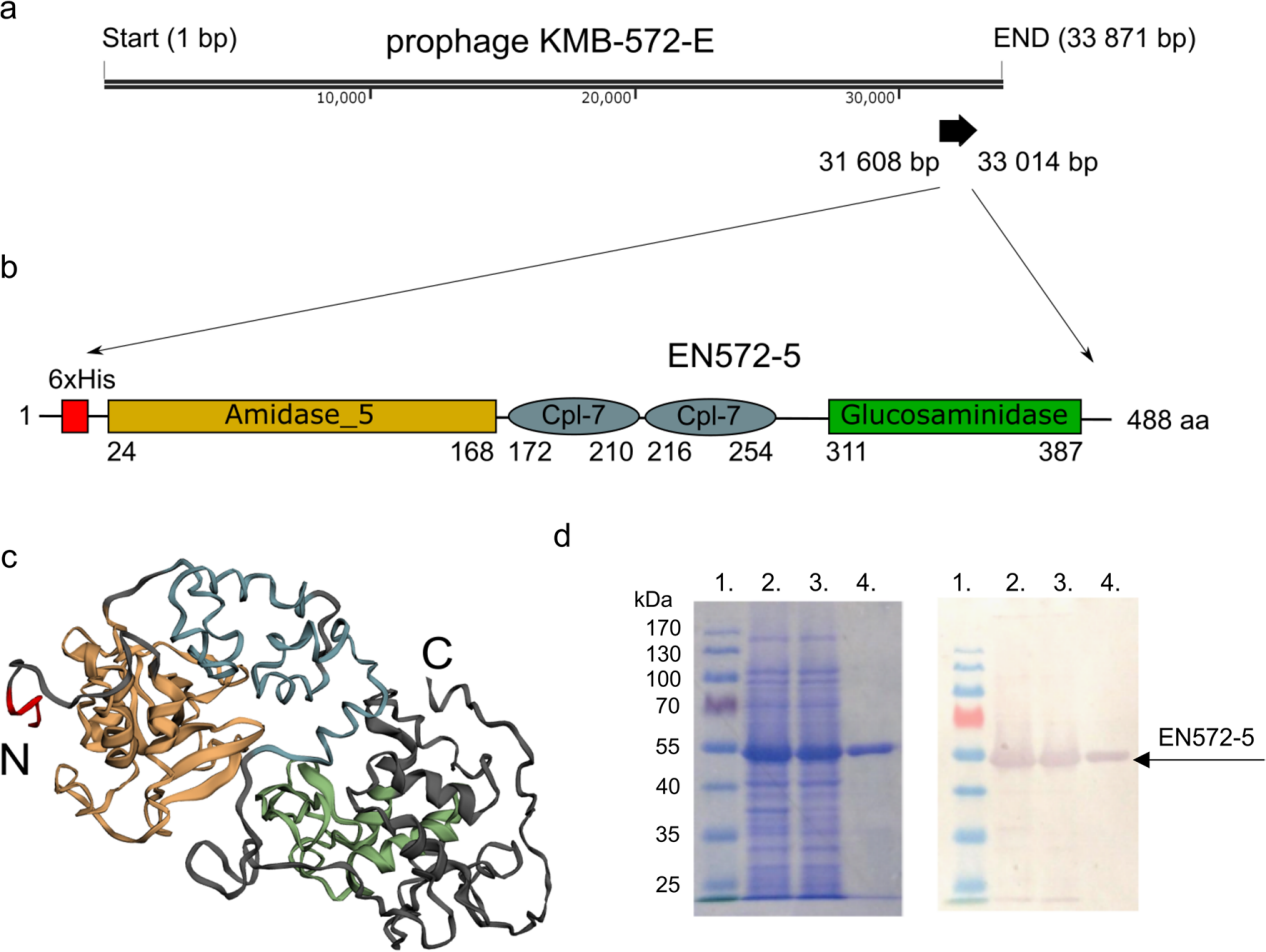
The in silico characterization and expression of endolysin EN572-5. (**a**) The genomic organization of EN572-5 in the genome of the prophage KMB-572-E. (**b**) A schematic representation of the locations of the catalytic (amidase_5, glucosaminidase) and binding (Cpl-7) domains of EN572-5. (**c**) A ribbon diagram of the predicted 3D structure of EN572-5 colored as follows: amidase_5 enzymatic domain (yellow), Cpl-7 binding motif domains (blue), glucosaminidase enzymatic domain (green), N-terminal His_6_Tag (red). (**d**) Analysis of recombinant EN572-5 (predicted molecular weight 52.9 kDa) by SDS-PAGE and Western blotting. Lane 1: Page Ruler Prestained protein Ladder (26616, Thermo Scientific). Lane 2: bacterial lysate containing EN572-5. Lane 3: soluble fraction of the bacterial lysate containing EN572-5. Lane 4: purified EN572-5

The predicted gene *EN572-5*, encoding a putative endolysin, was identified on the genome of the prophage KMB-572-E from *S. agalactiae* KMB-572 lysogen (Fig. 1a) (Lichvarikova et al. 2020). The corresponding protein EN572-5 consists of 468 amino acids with an expected molecular weight of 52.06 kDa. According to an NCBI CDD search, EN572-5 has two catalytic domains, an N-terminal amidase_5 domain (residues 24–168) belonging to the NLPC_P60 superfamily (cl21534; E-value 5.45 × 10^−82^) and a C-terminal glucosaminidase domain (residues 311–387) belonging to the Glucosaminidase superfamily (cl29459; E-value 6.90 × 10^−5^). In the protein mid-region, between the two catalytic domains, there are two Cpl-7 cell wall–binding domains (residues 172–210 and 216–254), from the CW_7 superfamily (cl07020; E-values 5.33 × 10^−13^ and 3.16 × 10^−4^, respectively) (Fig. 1b). The amidase_5 domain (N-acetylmuramoyl-l-alanine amidase) catalyzes the cleavage of a peptide bond between the d-glutamine and l-lysine of the peptidoglycan stem peptide; the glucosaminidase domain (Mannosyl-glycoprotein endo-β-N-acetylglucosamidase-like domain) cleaves the peptidoglycan sugar backbone (Pritchard et al. 2007; Donovan and Foster-Frey 2008).

A sequence alignment of EN572-5 by BLASTP showed that EN572-5 is identical to a peptidoglycan amidohydrolase from *Streptococcus* spp. (accession number WP_000405193), a lysin from the *S. agalactiae* prophage LambdaSa2%2C (accession number CFQ77715.1) and a lysin from *S. agalactiae* phage Javan33 (accession number QBX17128.1). A 3D model of EN572-5 was constructed using Phyre2 (Fig. 1c).

Recombinant EN572-5 was expressed in *E. coli* in soluble form. The protein was isolated at room temperature using nickel-ion affinity chromatography. About 5 mg of protein could be isolated from 50 mL of induced bacterial culture. The results of SDS-PAGE and Western blotting are shown in Fig. 1d. The size of the purified and immunodetected band of EN572-5 was 53 kDa, which corresponds to the predicted molecular weight of recombinant EN572-5 (52.9 kDa) (Fig. 1d). The presence of a His_6_Tag on the expressed protein was confirmed by Western blotting (Fig. 1d). EN572-5 was isolated as a soluble protein and could be highly purified (> 90%).

### Endolysin concentration study

**Fig. 2:**
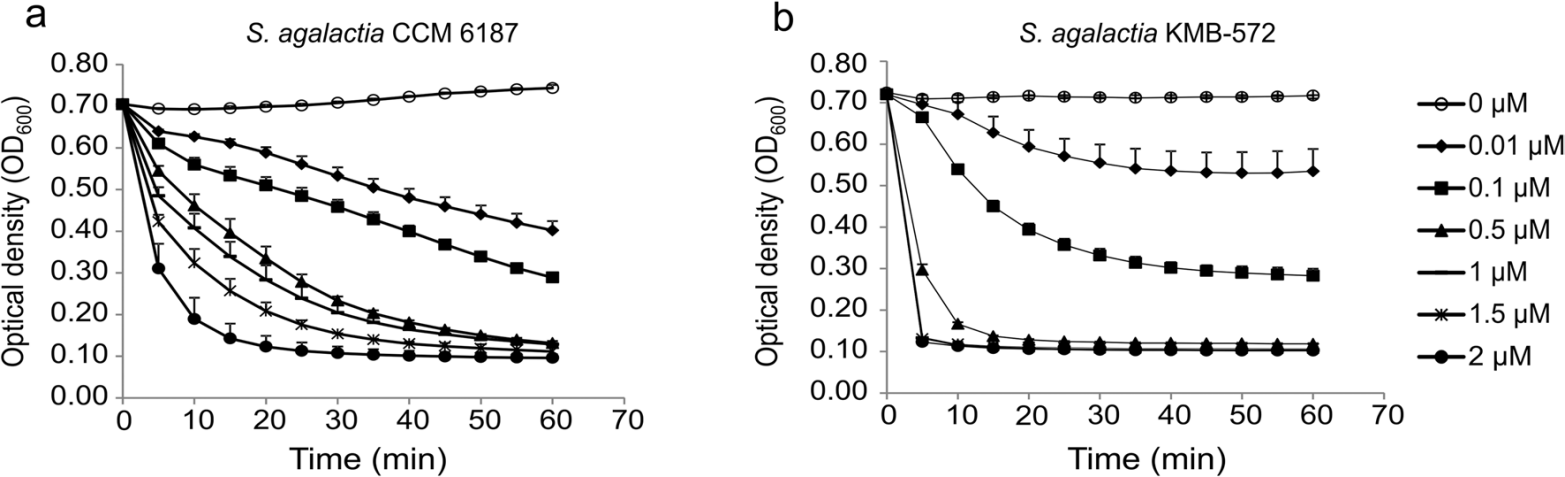
Lysis of (**a**) *S. agalactiae* CCM 6187 and (**b**) *S. agalactiae* KMB-572 in the presence of various concentrations of endolysin EN572-5. *Circle*: negative control (no endolysin added), *black diamond*: 0.01 µM enzyme added, *black square*: 0.1 µM enzyme added, *black triangle*: 0.5 µM enzyme added, *black line*: 1 µM enzyme added, *cross*: 1.5 µM enzyme added, and *black circle*: 2 µM enzyme added

As shown in Fig. 2, endolysin EN572-5 effectively lysed bacterial substrates containing the reference strain *S. agalactiae* CCM 6187 (Fig. 2a) and the clinical strain *S. agalactiae* KMB-572 (Fig. 2b) in a concentration range of 2 to 0.01 µM. At a concentration of 2 μM, the enzyme reduced the optical density of the reference strain from 0.7 to 0.1 (a 91% decrease) in 30 min (Fig. 2a). The optical density of the clinical strain was reduced from 0.7 to 0.1 in 5 min (Fig. 2b). The optimal protein concentration for further testing was determined to be 0.5 μM.

### Characterization of the lytic activity of EN572-5

**Fig. 3:**
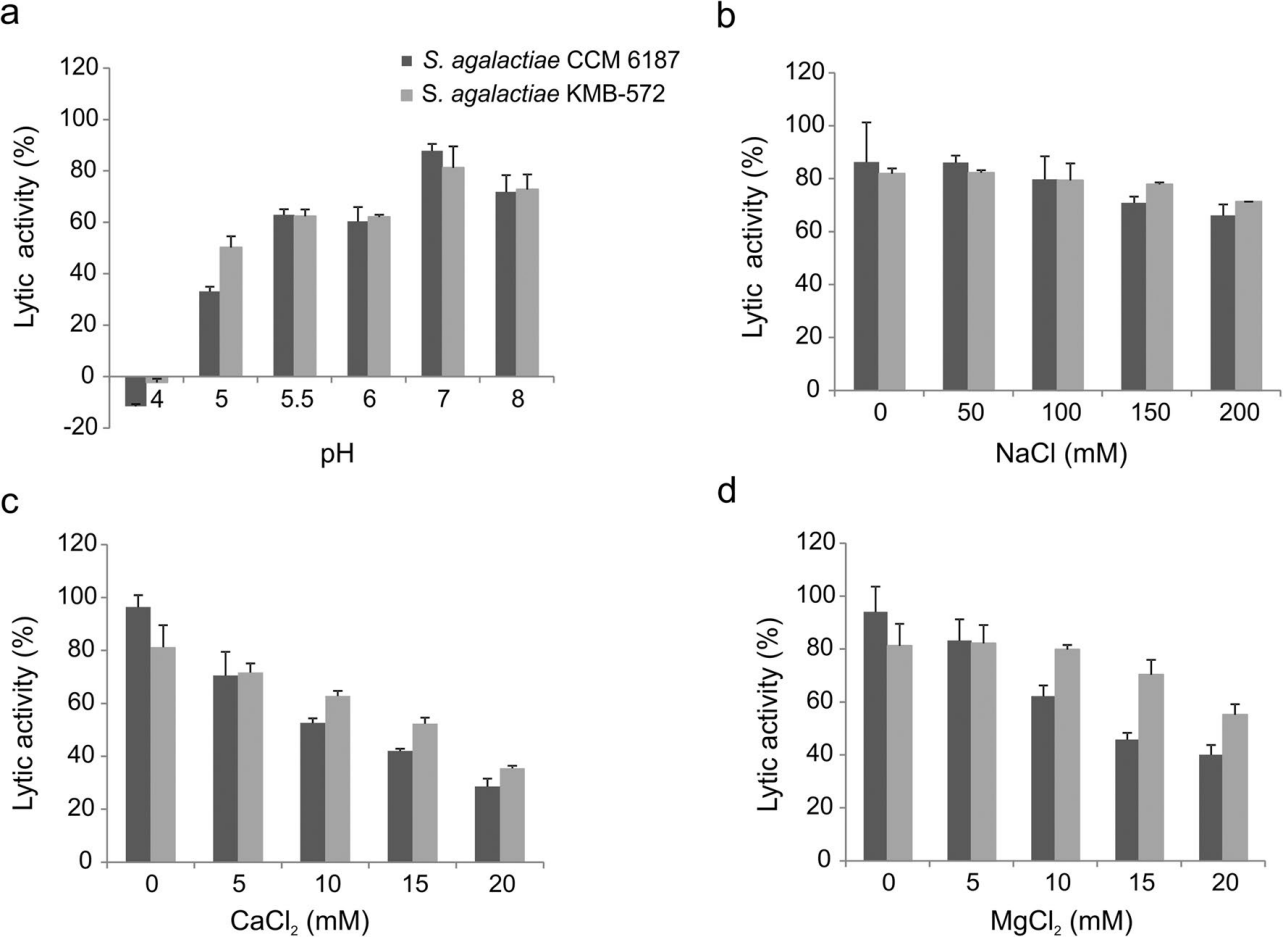
The effect of various factors on the lytic activity of EN572-5. The lytic activity of EN572-5 (0.5 µM) against *S. agalactiae* CCM 6187 (black column) and *S. agalactiae* KMB-572 (gray column) was measured at different (a) pH, and (b) NaCl (c) CaCl_2_, and (d) MgCl_2_ concentrations. Each column represents the mean of three experiments, and the *error bars* represent one standard deviation

*Effect of pH*: EN572-5 exhibited lytic activity at a pH range of 5.5–8.0 (62–87%) with the highest lytic activity detected at neutral pH (7.0). At pH 4.0, EN572-5 displayed no lytic activity (Fig. 3a).

*Effect of ions*: The lytic activity of EN572-5 was measured in 50 mM Tris–HCl, pH 7.0 supplemented with increasing concentrations of NaCl (0–200 mM), CaCl_2_ (0–20 mM), and MgCl_2_ (0–20 mM). NaCl induced no significant difference in lytic activity up to a concentration of 150 mM NaCl (Fig. 3b), but increasing concentrations of CaCl_2_ (Fig. 3c) and MgCl_2_ (Fig. 3d) decreased the lytic activity of EN572-5 by 46–68% and 26–54%, respectively.

**Fig. 4:**
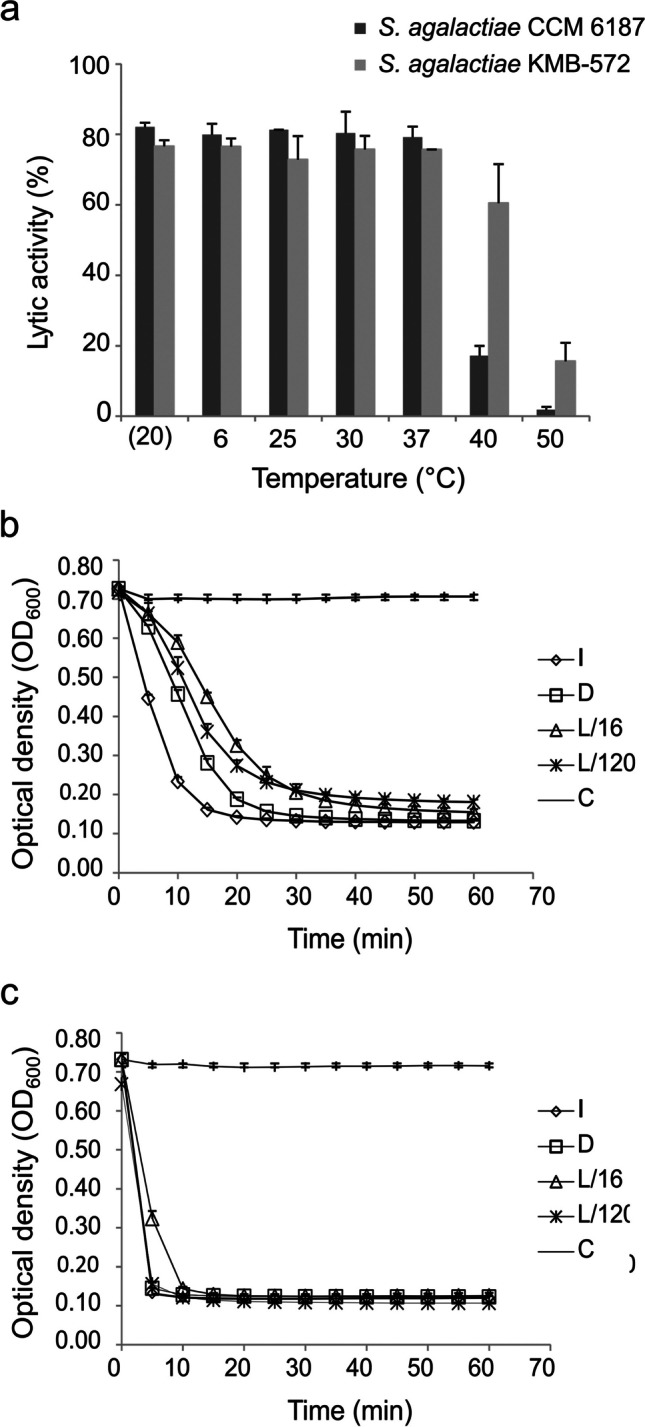
The effect of various temperatures and lyophilization on the lytic activity of EN572-5. (**a**) Lysis of *S. agalactiae* CCM 6187 (dark gray column) and *S. agalactiae* KMB-572 (gray column) with EN572-5 samples (0.5 µM) incubated at different temperatures for 30 min. (**b**) Lytic activity of EN572-5 against *S. agalactiae* CCM 6187 and (**c**) *S. agalactiae* KMB-572 with samples before and after lyophilization. *Diamond*: I – isolated enzyme, *square*: D – isolated enzyme after dialysis, *triangle*: L/16 – reconstituted enzyme 16 days after lyophilization, *cross*: L/120 – reconstituted enzyme 120 days after lyophilization, *line*: C – negative control (no endolysin added). The data shown are the mean value from three independent measurements and the *error bars* represent one standard deviation

*Effect of temperature*: The average lytic activity of EN572-5 after 30-min incubation at temperatures from − 20 to 37 °C was about 80% (Fig. 4a). After incubation at 40 °C, the lytic activity decreased to 61% on the clinical strain substrate and was only 17% on the reference strain. Overall, the lytic activity of EN572-5 decreased with increasing temperature above 37 °C and was only 2 − 16% at 50 °C (Fig. 4a). Given that the temperature of the human body is approximately 37 °C, the optimum temperature of EN572-5 taken to be 37 °C.

*Lyophilization*: For proteins with the potential to be used medically, verification of their stability under both long-term and dry conditions is necessary. EN572-5 was lyophilized and stored at − 20 °C for 16 days (L/16) and 120 days (L/120). After reconstitution, the lyophilized protein (both L/16 and L/120) reduced the optical density of the reference cell substrate from 0.7 to 0.15 in 1 h (75%), whereas a freshly isolated protein before lyophilization (I) reduced the same substrate to 0.15 in 15 min, and a protein after dialysis (D) did the same in 30 min (I, D 79%) (Fig. 4b). The lytic activity of EN572-5 was the same before lyophilization (I and D) and after reconstitution (both L/16 and L/120), when tested on a clinical strain substrate where the optical density was reduced from 0.7 to 0.12 in about 10–15 min (81%); stabilizing excipients were not used (Fig. 4c). In conclusion, the lytic activity of EN572-5 remained the same even 16 and 120 days after lyophilization and storage at − 20 °C.

### Lytic spectrum of EN572-5

**Fig. 5:**
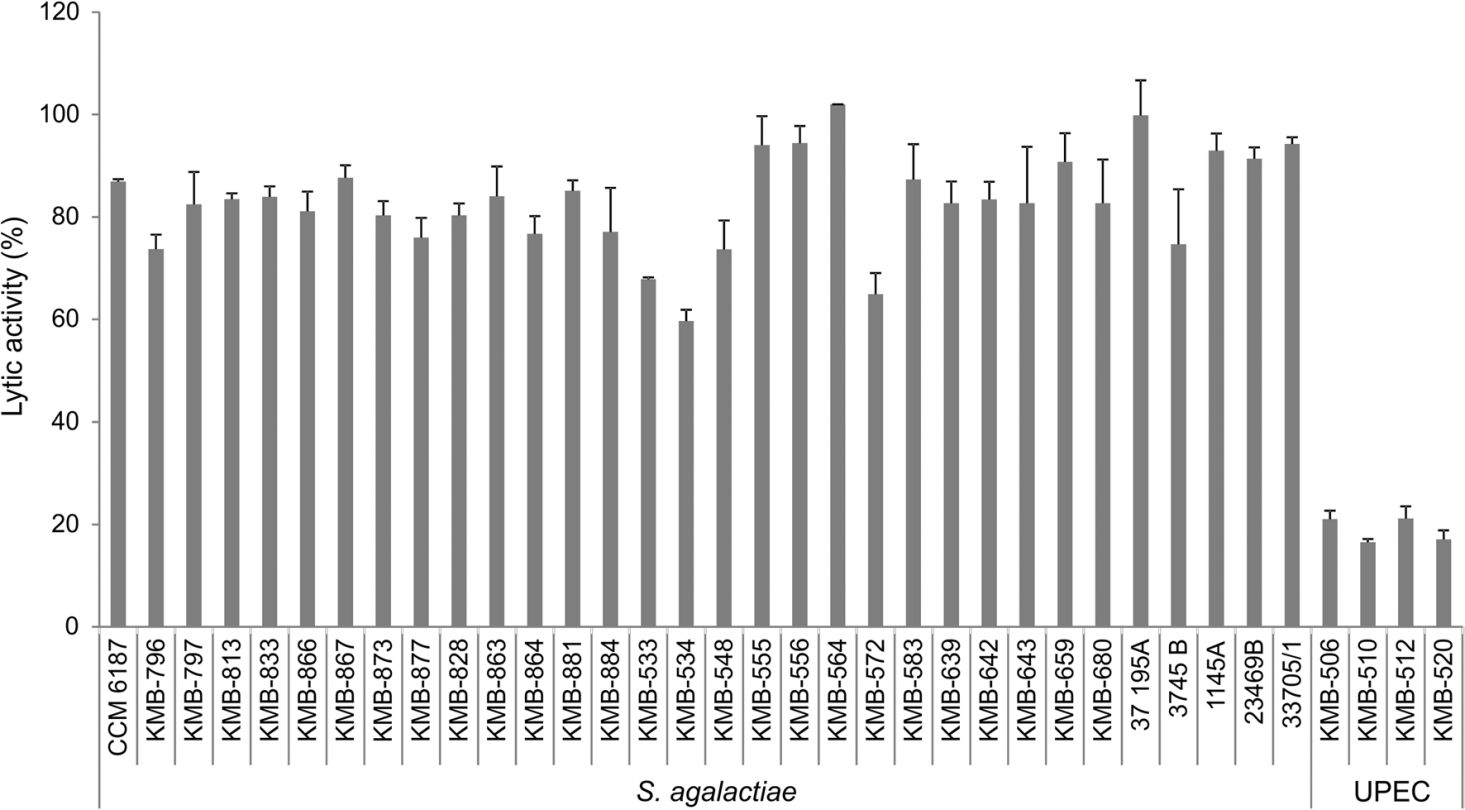
The lytic effect of EN572-5 against different *S. agalactiae* and UPEC strains. Cell suspensions of thirty-two *S. agalactiae* strains and four UPEC strains were treated with 0.5 μM amounts of EN572-5. The data shown are the mean values from three independent measurements, and the *error bars* represent one standard deviation

The lytic spectrum of EN572-5 was determined by turbidity reduction assays against thirty-two GBS strains, *S. dysgalactiae*,* S*. *mutans*, *S*. *pyogenes*, *S. salivarius*, *S*. *thermophiles*, *S*. *tigurinus*, *S*, *uberis*, and four UPEC strains (Table S1a). The resulting lytic activity was calculated using Eq. (1) of Oliveira et al. (2015). EN572-5 displayed lytic activity against all tested clinical GBS strains (human vaginal or urinal isolates) (Fig. 5). The lytic activity of EN572-5 ranged from 59.6% (on *S. agalactiae* KMB-534) to 100% (on *S. agalactiae* KMB-564) and the average antimicrobial activity was about 83%. There was no obvious correlation between lytic activity and the serotype or sequence type of the strains used. EN572-5 also displayed strong lytic activity against other *Streptococcus* spp. (average lytic activity of 74%) with the exceptions of *S*. *mutans* and *S*. *thermophilus* (Fig. S1). EN572-5 also showed 16–20% lytic activity against UPEC strains (Fig. 5). Cells were not pretreated with chloroform or EDTA. EN572-5 did not exhibit lytic activity against the tested *Lactobacillus* spp., however (Fig. S2).

#### Analysis of the *maeK* gene

An in silico analysis of twenty *S. agalactiae* genomes identified the presence of the *mae* gene cluster in all analyzed isolates (data not shown). Comparing the *maeK* gene of these twenty *S. agalactiae* strains with the same gene in *S. agalactiae* ABSA 1014 and *S. agalactiae* UPSA 807 (accession numbers KU061060 and KU061063) (Ipe et al. 2016) identified both an intact version of *maeK* and a version with a single base pair deletion. This deletion likely results in the production of a truncated protein using an alternative ATG start codon (it is expected to lack the first 17 amino acids) (Fig. S3a). The intact *mae*K gene was present in the genome of ten isolates while the remaining ten contained the version with the single base pair deletion. Serotype V was predominantly found in the UPSA isolates; only one isolate belonged to serotype VII. The serotype compositions of ABSA isolates were more variable and serotypes Ia, Ib, II, and III (Table S1b) were detected.

#### Growth of *S. agalactiae* in human urine

The clinical isolates ABSA KMB-797 and UPSA KMB-564 were selected for human urine growth assays based on an analysis of the *mae* gene cluster and on their different serotype and sequence types. The results of the assay are shown in Fig. S3b. ABSA strain KMB-797 showed massive growth in human urine. In contrast, UPSA strain KMB-564 grew only poorly and completely lost viability after 30 h (Fig. S3b). The average generation time of ABSA KMB-797 was 120 min (calculated between 0 and 24 h), while that of UPSA KMB-564 was 915 min.

### Lytic activity of EN572-5 in human urine

**Fig. 6:**
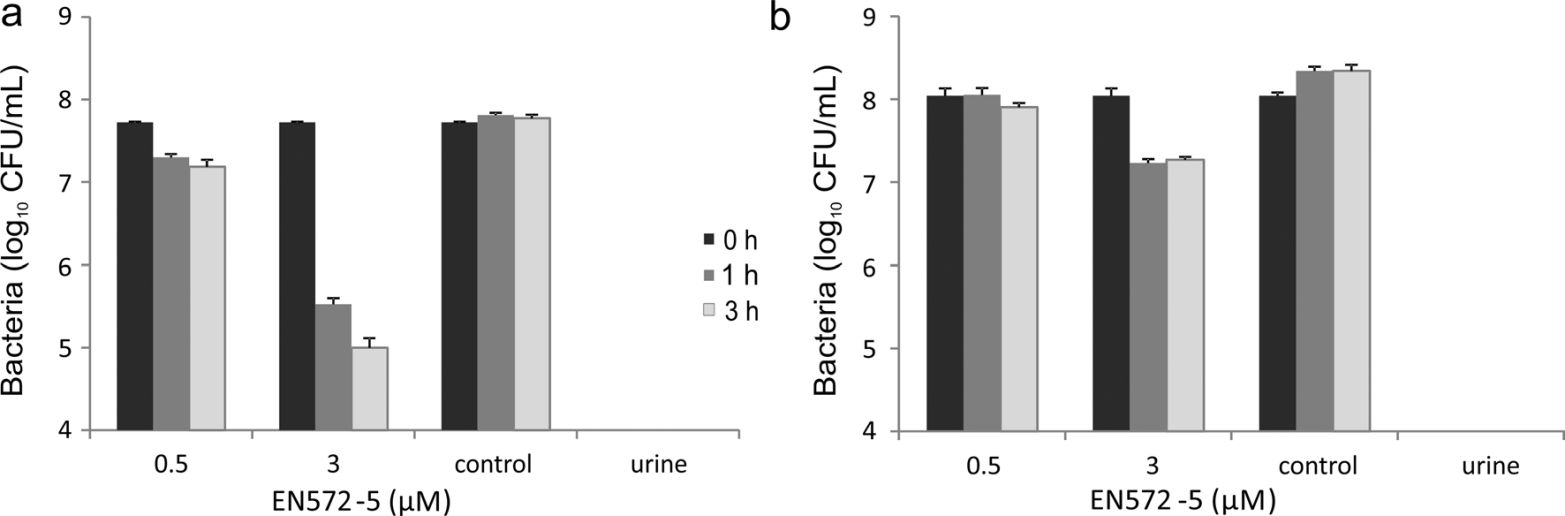
The inhibition of *S. agalactiae* growth in human urine by endolysin EN572-5. The bacteriostatic effect of EN572-5 (0.5 µM and 3 µM) was monitored in human urine against (**a**) UPSA KMB-564 and (**b**) ABSA KMB-797 and evaluated by colony counting after treatment for 1 h and 3 h. The data shown are the mean values from three independent measurements, and the *error bars* represent one standard deviation

The antibacterial potential of EN572-5 against *S. agalactiae* during UTI was investigated in human urine using the colony counting method (Fig. 6). The lytic activity of EN572-5 against UPSA KMB-564 was measured at concentrations of 0.5 μM and 3 μM. Endolysin EN572-5, at a final concentration of 3 μM, was more effective in eliminating UPSA KMB-564 in urine. An initial infection dose of 4 × 10^7^ CFU/mL decreased to about 7.3 × 10^5^ CFU/mL in 1 h, and to 1.4 × 10^5^ CFU/mL in 3 h (Fig. 6a). EN572-5 at a final concentration of 0.5 μM, decreased the initial UPSA infection dose to about 3.0 × 10^6^ CFU/mL in 1 h and still had approximately the same value after 3 h. In contrast, EN572-5 had a lower lytic effect against ABSA KMB-797 at both concentrations (0.5 μM and 3 μM). Little difference was noted at 0.5 μM and the 3 μM concentration decreased the initial infection dose of ABSA KMB-797 from 1.3 × 10^8^ CFU/mL to about 2.3 × 10^7^ CFU/mL after 1 h and was nearly the same after 3 h (Fig. 6b).

## Discussion

UTIs caused by GBS are relatively rare (Tan et al. 2012; Foxman 2010), but are dangerous for pregnant women, elderly patients, and immunosuppressed individuals (Sullivan et al. 2017). Some individuals suffer from recurrent UTI and therefore they are repeatedly treated with antibiotics. Frequent treatment with antibiotics results in an increase in resistance and antibiotic treatment becomes ineffective. At the same time, the repeated use of antibiotics can result in the disruption of an individual’s healthy vaginal or intestinal microbiome, potentially leading to the development of secondary diseases. These two considerations make it necessary to search for alternative treatments for infections caused by resistant bacteria.

The therapeutic potential of several recombinant endolysins in the treatment of group B streptococcal infections has been verified in several studies (Doehn et al. 2013; Gilmer et al. 2013; McCullers et al. 2007; Loeffler et al. 2001). Compared with antibiotics, endolysins have many advantages, such as non-proliferation, fast bactericidal activity, a wide host spectrum, definite pharmacokinetics, and a low possibility for resistance development (Pastagia et al. 2011).

One potential concern with endolysin treatment is an adverse immune response. Early studies confirmed that, although endolysins are immunogenic, antibodies against the corresponding endolysins do not remarkably diminish their lytic activity in vitro. For example, the bactericidal activity and binding capacity of the staphylococcal-specific endolysin LysGH15 were not blocked even after incubation with anti-LysGH15-serum for 60 min (Zhang et al. 2016). Furthermore, experiments with the pneumococcal-specific endolysin Cpl-1 in immunized rabbit serum (in vitro) and immunized mice (in vivo) did not affect its therapeutic efficacy (Loeffler et al. 2003). Also, to the best of our knowledge, no case of bacterial resistance to endolysins has been reported. Even repeated exposure of staphylococci, pneumococci, and *B. cereus* to low concentrations of endolysins does not spontaneously generate resistant mutants (Loeffler et al. 2001; Schuch et al. 2002).

The main aim of the present study was to isolate and characterize a newly prepared endolysin, EN572-5, derived from the prophage KMB-572-E region of the human clinical isolate *S. agalactiae* KMB-572 (Fig. 1a) (Lichvarikova et al. 2020). *S. agalactiae* strains often contain prophages in their genome, which also include many endolysins (Pritchard et al. 2004). A sequence identical to EN572-5 is found as a peptidoglycan amidohydrolase protein (WP_000405193), in the genomes of several streptococcal prophages, including LambdaSa2%2C (CFQ77715.1) and Javan33 (QBX17128.1).

EN572-5 has a three-domain structure composed of two terminal catalytic domains, amidase_5 and glucosaminidase, and two central Cpl-7 binding domains, in tandem (Fig. 1b). The same sort of domain organization is also found in streptococcal endolysins λSa2 and PlySK1249. Indeed, this kind of modular architecture, in which two different types of functional domains are jointly linked to a single CBD, is especially common in staphylococcal and streptococcal endolysins (Becker et al. 2009).

The gene encoding endolysin EN572-5 was cloned, expressed as recombinant protein with an N-terminal His_6_Tag, and purified. Optimal reaction and storage conditions of the isolated recombinant EN572-5 were determined, the lytic activity of this endolysin was tested against several *Streptococcus* spp*.* strains, and its ability to eliminate GBS in human urine in a UTI model was verified.

We also tested the effects of different factors on EN572-5 lytic activity. Microbial colonization of the urinary tract depends on several features such as pH, oxygen tension, osmolarity, nutrient availability, adhesion sites, and immune system interactions (Gritz and Bhandari 2015). The pH of human urine varies between individuals and can range from 4.5 to 8.6 (Neugent et al. 2020). The optimal pH conditions for GBS growth are from 6.0 to 7.0. GBS bacteria survive poorly at low pH and even fail to grow at pH 4.3 (Yang et al. 2012). The optimal pH for EN572-5’s lytic activity was determined to be 7.0, and it was highly active against a clinical isolate (above 50%) in the pH range of 5.0 to 8.0; at pH 4.0, it displayed no lytic activity, however (Fig. 3a). Other endolysins active against streptococcal strains have various optimal pH values, for example, endolysin PlyGBS from 4.0 to 6.0 (Cheng et al. 2005), lysin B30 from 4.5 to 6.0 (Pritchard et al. 2004), and endolysin PlySK1249 from 7.0 to 8.5 (Oechslin et al. 2013).

The optimal reaction temperature of EN572-5 was determined to be 37 °C (Fig. 4a), similar to human urine temperature, which ranges from 36.9 to 39.9 °C, and body temperature, which averages 37 °C (Neugent et al. 2020).

Human urine is composed of many soluble elements, including electrolytes, osmolytes, amino acids, and carbohydrates (Neugent et al. 2020; Reitzer and Zimmern 2019). Divalent metal ions often bind to amino acid residues in endolysin domains, thereby altering their lytic activity (Son et al. 2012; Jumpei et al. 2018). For example, the streptococcal phage Ply700 endolysin exhibited rapid, calcium-dependent lysis against *S. uberis*, *S. pyogenes*, and *S. dysgalactiae* while showing only slight lysis activity toward *S. agalactiae* (Celia et al. 2008). Calcium ions also enhanced the activity of the streptococcal enzyme EN534-C (Bocanova et al. 2022), and the chimeric lysin ClyC against *S. aureus* strains (Li et al. 2021) and LysGH15 (Gu et al. 2014). In the present case, we found that NaCl did not have a significant effect on EN572-5 activity (Fig. 3b) and that increasing concentrations of CaCl_2_ (Fig. 3c) and MgCl_2_ (Fig. 3d) actually decreased EN572-5 lytic activity (Fig. 3c, d), especially against the reference strain. However, EN572-5 still showed significant lytic activity on the clinical strain KMB-572, over 35%, in the presence of 20 mM CaCl_2_ and 40% in the presence of 20 mM MgCl_2_. The difference between EN572-5 and the previously studied proteins is likely due to their different functional domains. EN572-5 contains a amidase_5 domain while all the previously studied proteins possess a CHAP catalytic domain.

Enzymes intended for use as therapeutics need to be stable under both long-term and dry conditions. One option for the long-term storage of endolysins is lyophilization (Carpenter et al. 1997). However, not all lytic proteins retain their activity after rehydration. Excipients such as BSA and sugars are regularly added to prevent proteins from denaturing during freeze-drying. Endolysin EN572-5 was able to maintain its antibacterial activity (81%) against the clinical strain 120 days after lyophilization and storage at − 20 °C without any stabilizing excipients (Fig. 4b, c). This indicates that EN572-5 could be a good candidate for further development into a drug for the treatment of infections caused by streptococci. Similar stability after lyophilization was reported for the chimeric staphylococcal endolysin ClyC (Li et al. 2021).

The lytic activity of EN572-5 against selected human clinical isolates of GBS (Table S1a) was tested. All tested strains (serotypes I–VII) were highly (~ 83%) susceptible to lysis by endolysin EN572-5 (Fig. 5). Of the best characterized streptococcal endolysins, only PlyGBS had previously shown significant lytic activity against GBS (Cheng et al. 2005). Endolysin λSa2 also exhibited lytic activity against *S. agalactiae*, *S. pneumoniae*, and *S. aureus* (Pritchard et al. 2007; Donovan and Foster-Frey 2008). We also determined the antibacterial potential of EN572-5 against several *Streptococcus* spp. All streptococci strains were lysed except for *S. mutans* and *S. thermophilus* (Fig. S1).

The *E. coli* Gram-negative bacteria still causes the vast majority of UTIs (Flores-Mireles et al. 2015) and endolysins usually show low activity against Gram-negative bacteria because their outer membrane effectively presents a physical protective barrier that prevents exogenous lysozyme from reaching the peptidoglycan substrate (Lai et al. 2011; Yang et al. 2020). As expected, EN572-5 had only a moderate lytic effect against selected UPEC strains (Fig. 5). Despite its broad lytic spectrum against different STs of GBS and other *Streptococcus* spp., EN572-5 showed no lyses of lactobacilli, beneficial vaginal commensal flora (Fig. S2). Compared to antibiotics, this is a great benefit. After treating UTI with antibiotics, secondary infections often occur, such as vaginosis. Lactobacilli in the vagina are thought to be a key defensive mechanism against infection and can accelerate the re-epithelialization of vaginal epithelial cells (Liu et al. 2023b).

Ipe et al. (2016) found that not all GBS can grow in urine. Based on this ability, GBS are divided into ABSA and UPSA strains; ABSA can grow efficiently in human urine, while UPSA cannot. In ABSA, a functionally intact ME pathway (*mae* gene cluster), especially the product of the *maeK* gene, is required for robust growth in human urine by providing the ability to catabolize malic acid (Ipe et al. 2016). Our in silico analysis of the *mae* gene cluster of twenty *S. agalactiae* genomes confirmed the presence of an intact version (10 isolates) and a truncated version (10 isolates) of the MaeK protein (Fig. S3a). The presence of this protein was also correlated with the ability of these strains to grow in human urine (Fig. S3b).

When at least 10^5^ CFU/mL of GBS are present in the urine, with or without symptoms of a urinary tract infection, it is necessary to start treatment; otherwise, the infection can develop into kidney damage or to preterm labor (Rosenberger et al. 2020; Henderson et al. 2019). The antibacterial potential of EN572-5 was investigated in human urine against human clinical isolates ABSA (KMB-797) and UPSA (KMB-564), where we observed differences its bacteriolytic effect (Fig. 6). EN572 was more effective against the UPSA isolate (2.77 log_10_ reduction) over a 3 h incubation at 37 °C than against the ABSA isolate (0.8 log_10_ reduction). These differences could correlate with the ability of these strains to grow in urine, or with different serotypes or with their overall sensitivity to EN572-5. However, UPSA, unlike ABSA, adheres to and invades human bladder urothelial cells more efficiently, colonizes the bladder rapidly, kills urothelial cells more effectively, and induces a more rapid and robust pro-inflammatory cytokine response in human urothelial cells (Leclercq et al. 2016).

In conclusion, endolysin EN572-5, derived from human clinical isolate *S. agalactiae* KMB-572, was cloned, isolated, and characterized. We described for the first time the lytic activity of a streptococcal endolysin in human urine, using an in vitro model of UTI caused by GBS. Endolysin EN572-5 showed a bacteriolytic effect against UPSA and a bacteriostatic effect against ABSA in human urine. Recombinant EN572-5 has the potential to become an antimicrobial agent for the treatment of *S. agalactiae* infections, though additional experiments will be needed to investigate its antibacterial potential in human urine.

## Supplementary Information

Below is the link to the electronic supplementary material.

Supplementary file1 (PDF 576 KB)

## Data Availability

The datasets generated during and/or analyzed during the current study are available from the corresponding author on reasonable request.
