# Supplementary material for: Endolysin EN572-5 as an alternative to treat urinary tract infection caused by Streptococcus agalactiae
Source: Appl Microbiol Biotechnol. 2024 Jan 8;108(1):79. doi: 10.1007/s00253-023-12949-8 (PMC10774192; doi:10.1007/s00253-023-12949-8)
Supplement: Supplementary file 1 — Supplementary file1 (PDF 576 KB) [file 253_2023_12949_MOESM1_ESM.pdf]

Title Endolysin EN572-5 as an alternative to treat urinary tract infection caused by *Streptococcus agalactiae*

Applied Microbiology and Biotechnology, Springer
Maria Kajsikova, Michal Kajsik, Lucia Bocanova, Kristina Papayova, Hana Drahovska and Gabriela Bukovska*

***Correspondence**

Gabriela Bukovska
Gabriela.Bukovska@savba.sk
Department of Genomics and Biotechnology, Institute of Molecular Biology SAS, Dubravska cesta 21, 845 51 Bratislava, Slovakia

Table S1a Bacterial strains used in this study

Strain	Serotype	ST	Source	Lytic activity (%)
SA KMB-533	V	ST-1	urine	67.84
SA KMB-534	III	ST-17	urine	59.63
SA KMB-548	III	ST-19	vaginal swab	73.64
SA KMB-555	Ia	ST-23	urine	94,01
SA KMB-556	V	ST-1	urine	94,41
SA KMB-564	V	ST-1402	urine	102,01
SA KMB-572	VII	ST-130	vaginal swab	64.88
SA KMB-583	III	ST-19	urine	87.30
SA KMB-639	Ib	ST-6	vaginal swab	82.69
SA KMB-642	Ia	ST-23	urethral swab	83.40
SA KMB-643	IV	ST-291	urine	82.66
SA KMB-659	II	ST-12	urine	90.77
SA KMB-680	II	ST-22	urine	82.67
SA KMB-796	III	ST-17	vaginorectal swab	73.73
SA KMB-797	Ia	ST-23	vaginorectal swab	82.44
SA KMB-813	VI	ST-1	vaginal swab	83.48
SA KMB-828	III	ST-17	vaginal swab	80.30
SA KMB-833	V	ST-1401	vaginal swab	83.94
SA KMB-863	V	ST-498	vaginal swab	84.03
SA KMB-864	II	ST-12	vaginal swab	76.70
SA KMB-866	Ib	ST-6	vaginorectal swab	81.12
SA KMB-867	Ib	ST-8	vaginorectal swab	87.64
SA KMB-873	IV	ST-459	vaginorectal swab	80.32
SA KMB-877	II	ST-12	vaginorectal swab	75.94
SA KMB-881	II	ST-12	vaginorectal swab	85.13
SA KMB-884	III	ST-17	vaginorectal swab	77.08
SA 37 195A	-	-	-	99.81
SA 3745 B	-	-	-	74.66
SA 1145A	-	-	-	92.97
SA 23469B	-	-	-	91.36
SA 33705/1	-	-	-	94.26
SA CCM 6187	-	-	bovine type	86.90
S. mutans KMB-565	-	-	-	no lytic activity
S. pyogenes	-	-	-	77.88
S. salivarius KMB-593	-	-	-	82.33
S. thermophiles KMB-595	-	-		3.97

Table S1a Bacterial strains used in this study (continuation)

Strain	Serotype	ST	Source	Lytic activity (%)
S. tigurinus KMB-655	-	-	-	72.53
S. uberis KMB-594	-	-	-	72.82
S. dysgalactiae 23855B	-	-	-	67.66
S. dysgalactiae 21029C	-	-	-	71.69
UPEC KMB-506	-	-	-	21.6
UPEC KMB-510	-	-	-	16.54
UPEC KMB-512	-	-	-	21.17
UPEC KMB-520	-	-	-	17.13
L. crispatus 17A	-	-	-	no lytic activity
L. gasseri 13C	-	-	-	no lytic activity
L. jensenii 31A	-	-	-	no lytic activity
Gynimun Intim Care (Onapharm, CR)	-	-	-	no lytic activity

Table S1b The prevalence of serotypes in ABSA and UPSA isolates

Strain	Serotype	ST	ABSA/UPSA
SA KMB-533	V	ST-1	UPSA
SA KMB-562	V	ST-1	
SA KMB-564	V	ST-1	
SA KMB-572	VII	ST-130	
SA KMB-679	V	ST-1	
SA KMB-682	V	ST-1	
SA KMB-833	V	ST-1401	
SA KMB-861	V	ST-1401	
SA KMB-885	V	ST-1	
SA KMB-889	V	ST-1	
SA KMB-548	III	ST-19	ABSA
SA KMB-642	Ia	ST-23	
SA KMB-797	Ia	ST-23	
SA KMB-534	III	ST-17	
SA KMB-884	III	ST-17	
SA KMB-639	Ib	ST-6	
SA KMB-659	III	ST-12	
SA KMB-675	II	ST-12	
SA KMB-877	II	ST-12	
SA KMB-890	II	ST-10	

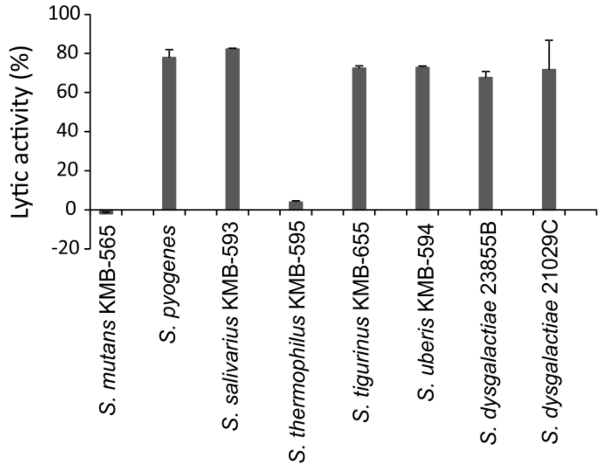

Fig. S1 The lytic effect of EN572-5 against *Streptococcus* spp. Cell suspensions of eight *Streptococcus* spp. strains were treated with 0.5 μM amounts of EN572-5. The data shown are the mean values from three independent measurements, and the error bars represent one standard deviation.

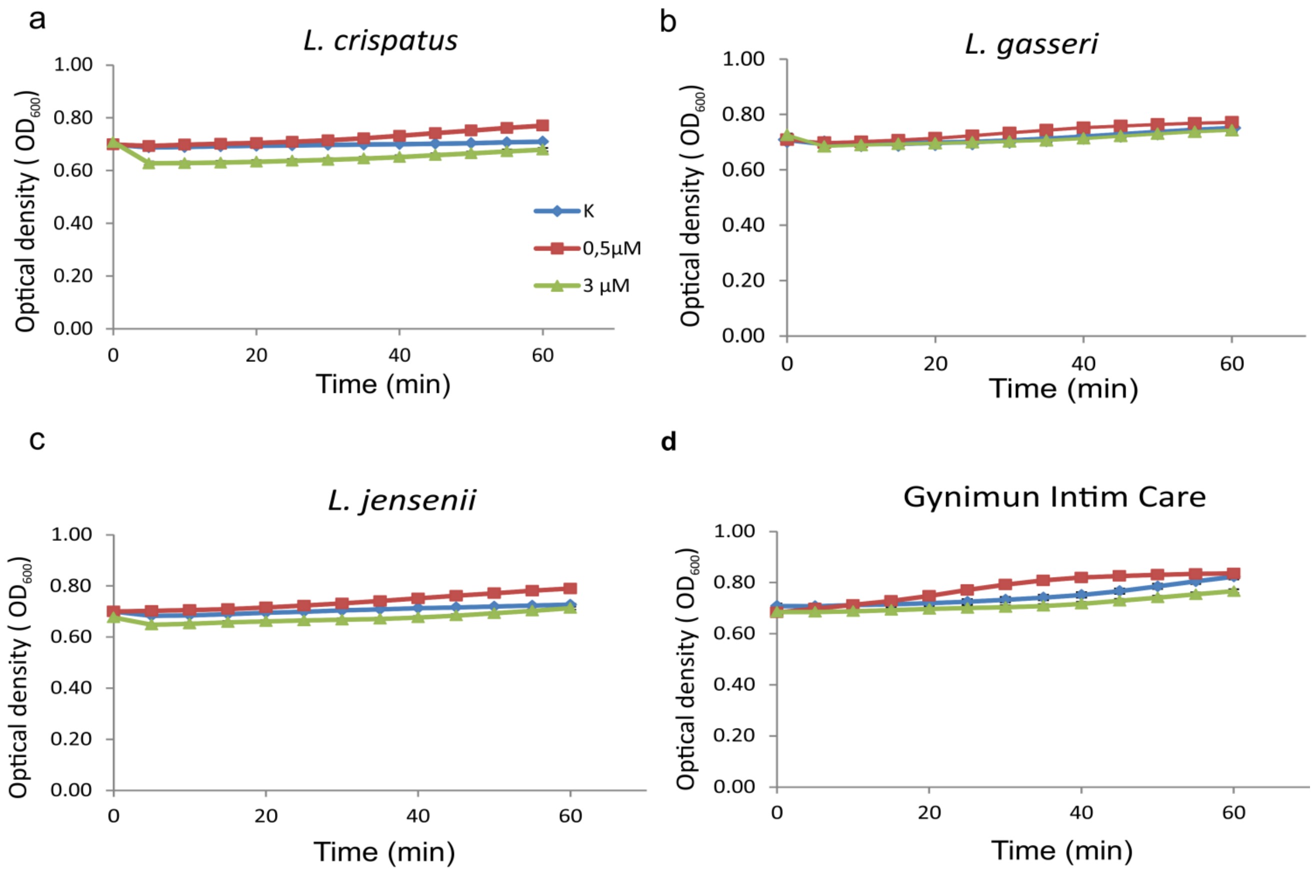

Fig. S2 The lytic effect of endolysin EN572-5 against *Lactobacillus* spp. Cell suspensions of three *Lactobacillus* spp. and one probiotic preparation with lactobacilli were treated with 0.5 μM and 3 μM amounts of EN572-5. The data shown are the mean values from three independent measurements, and the error bars represent one standard deviation.

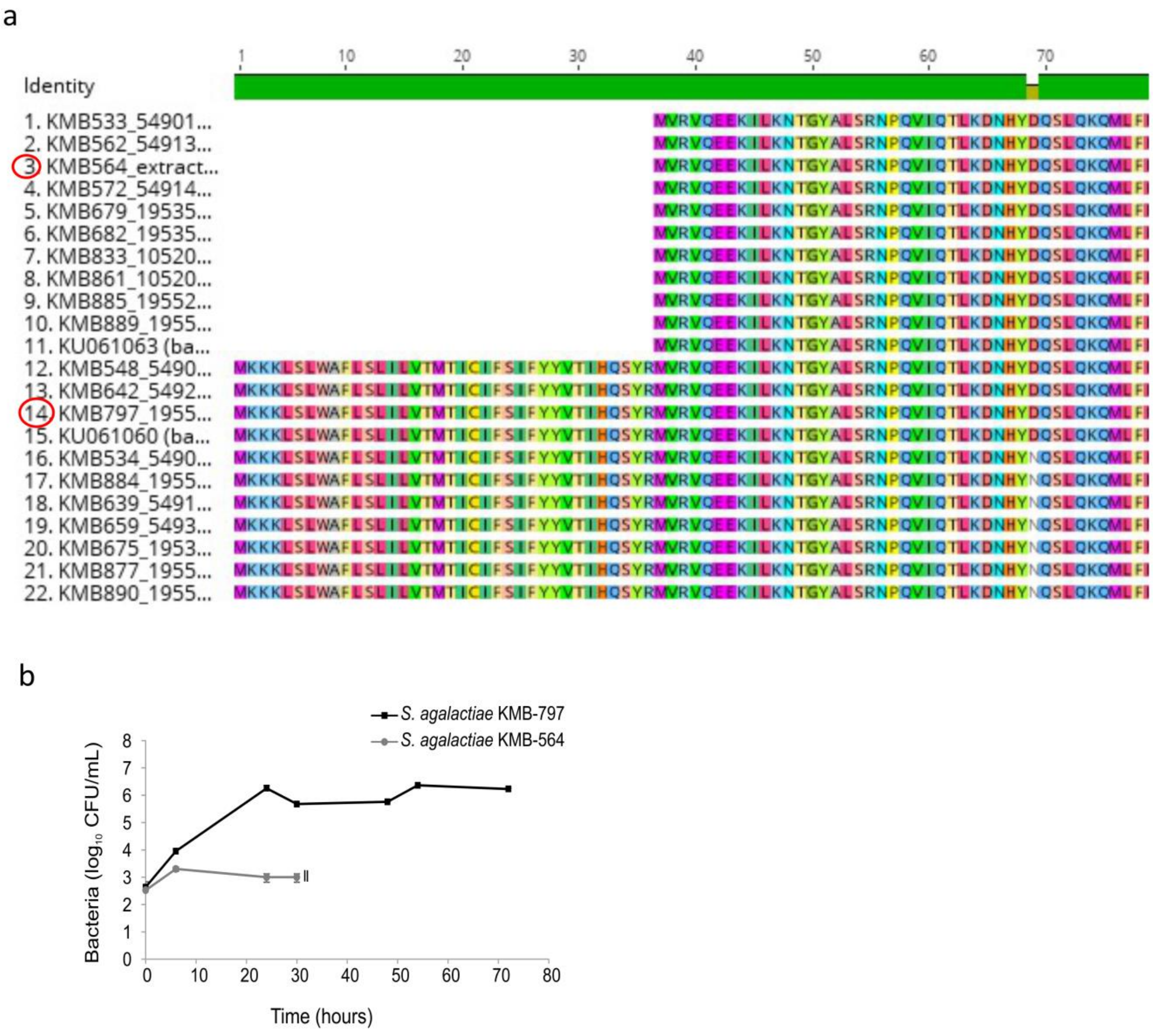

Fig. S3 (a) Comparison of the *maeK* gene product of twenty *S. agalactiae* strains with the *S. agalactiae* ABSA 1014 and *S. agalactiae* UPSA 807 strains. (b) The robust growth of ABSA KMB-797 (black square) in urine contrasts with the poor growth of UPSA KMB-564 (grey circle).
